# English Dentists

**Published:** 1895-06

**Authors:** 


ENGLISH DENTISTS.—There are 4874 registered dentists in Great Britain, of whom 3479 practice with no special qualification, but on the strength of their own declaration that they were engaged in the practice of dentistry before the passing of the recent act regulating the profession.

